# Physical activity protects NLRP3 inflammasome‐associated coronary vascular dysfunction in obese mice

**DOI:** 10.14814/phy2.13738

**Published:** 2018-06-21

**Authors:** Jonghae Lee, Yang Lee, Emily C. LaVoy, Michihisa Umetani, Junyoung Hong, Yoonjung Park

**Affiliations:** ^1^ Laboratory of Integrated Physiology Department of Health and Human Performance University of Houston Houston Texas; ^2^ Texas A&M Health Science College of Medicine College Station Texas; ^3^ Department of Biology and Biochemistry University of Houston Houston Texas

**Keywords:** Diet‐induced obesity, inflammation, vascular function, voluntary running

## Abstract

Activation of the nucleotide‐binding oligomerization domain‐like receptor family pyrin domain‐containing 3 (NLRP3) inflammasome mediates the release of pro‐inflammatory cytokine interleukin (IL)‐1*β* and thereby plays a pivotal role in the inflammatory response in vascular pathology. An active lifestyle has beneficial effects on inflammation‐associated vascular dysfunction in obesity. However, it remains unclear how physical activity regulates NLRP3 inflammasome‐mediated vascular dysfunction in obesity. Therefore, we explored the protective effect of physical activity on NLRP3 inflammasome‐associated vascular dysfunction in mouse hearts, and the potential underlying mechanisms. C57BL/6J male mice were randomly divided into four groups: (1) control low‐fat diet (LF‐SED), (2) LF diet with free access to a voluntary running wheel (LF‐RUN), (3) high‐fat diet (HF‐SED; 45% of calories from fat), and (4) HF‐RUN. We examined NLRP3 inflammasome‐related signaling pathways, nitric oxide (NO) signaling, and oxidative stress in coronary arterioles to test effects of HFD and physical activity. Voluntary running reduced NLRP3 inflammasome and its downstream effects, caspase‐1 and IL‐1*β* in coronary arteriole endothelium of obese mice in immunofluorescence staining. HF‐RUN attenuated HFD‐dependent endothelial NO synthase (eNOS) reduction and thus increased NO production compared to HF‐SED. HFD elevated intracellular superoxide production in coronary arterioles while voluntary running ameliorated oxidative stress. Our findings provide the first evidence that voluntary running attenuates endothelial NLRP3 inflammasome activation in coronary arterioles of HFD feeding mice. Results further suggest that voluntary running improves obesity‐induced vascular dysfunction by preserved NO bioavailability via restored expression of eNOS and reduced oxidative stress.

## Introduction

Obesity is a leading risk factor for cardiovascular disease (CVD) mortality and morbidity (Van Gaal et al. 2006), independently associated with the progression of coronary artery disease (Al Suwaidi et al. 2001). Excess caloric intake and physically inactive lifestyles are considered as important factors to contribute to obesity (Hill et al. 2012). Emerging evidence and our previous study suggest that primordial prevention is a more effective strategy to prevent the development of CVD that may begin in childhood and continue for decades (McGill and McMahan 1998; Weintraub et al. 2011; Park et al. 2012; Gillman 2015).

Endothelial dysfunction, mainly affected by reduced nitric oxide (NO) bioavailability and increased oxidative stress, is an important predictor of CVD. NO is a key element of vascular homeostasis to regulate vascular tone, tissue blood flow, and inflammatory responses (Sena et al. 2013). In obesity, nicotinamide adenine dinucleotide phosphate (NADPH) oxidase, NOX, family‐derived superoxide ($O_{2}^{\cdot -}$) generation (Konior et al. 2014) impairs endothelium‐dependent vasodilation by reduced endothelial NO synthase (eNOS)‐mediated NO formation (Sena et al. 2013). Chronic low‐grade inflammation is a hallmark in obesity that contributes to the development of CVD (Hotamisligil 2006). Increased expression of proinflammatory cytokines, including tumor necrosis factor‐alpha, interleukin‐6 (IL‐6), and IL‐1*β*, induces reactive oxygen species (ROS) and results in vascular endothelial dysfunction (Wassmann et al. 2004; Gao et al. 2007).

Inflammasomes have been highlighted as a pivotal player in obesity‐induced vascular pathophysiology (Liu et al. 2015; Wang et al. 2016a). The most widely studied inflammasome is the nucleotide‐binding oligomerization domain‐like receptor (NLR) family pyrin domain‐containing 3 (NLRP3) inflammasome, consisting of three distinct protein molecules: NLRP3, an apoptosis‐associated speck‐like protein containing (ASC), and caspase‐1. Diverse metabolic stimulators activate the NLRP3 inflammasome, subsequently cleaving active caspase‐1. Elevated caspase‐1 activity, in turn may lead to endothelial dysfunction and injury via its pyroptosis and proteolytic actions (Zhang et al. 2015; Xi et al. 2016) and through IL‐1*β* processing and secretion responsible for tissue inflammation, insulin resistance (Jager et al. 2007), and atherosclerosis pathogenesis (Duewell et al. 2010). Multiple studies using NLRP3 inflammasome inhibitors and genetic manipulation have provided further evidence of the critical roles of NLRP3 inflammasome in regulating CVD risk factors and endothelial dysfunction (Shao et al. 2015; Zhang et al. 2015; Wang et al. 2016a).

Regular physical activity is commonly prescribed to reduce the risk of obesity‐associated cardiovascular pathologies (Gielen et al. 2010). Interestingly, our previous study reported the protective effects of physical activity coinciding with high‐fat diet (HFD) on obesity‐associated vascular dysfunction of a murine model (Park et al. 2012). We showed that the primary mechanisms for exercise‐induced improvement of vascular function were enhanced eNOS‐mediated NO production and reduced oxidative stress and inflammatory signaling in vascular endothelium (Lee et al. 2011; Park et al. 2012). Furthermore, recent studies reported that chronic exercise training suppressed activation of NLRP3 inflammasome in the adipose tissue of obese mice (Mardare et al. 2016) and in the brain of ovariectomized mice (Wang et al. 2016b). However, the effect of physical activity on NLRP3 inflammasome activation‐associated vascular function in obesity is not tested elsewhere.

Therefore, the purpose of this study was to elucidate whether physical activity prevents the obesity‐induced NLRP3 inflammasome activation in coronary arterioles, and to investigate the underlying mechanism by which physical activity protects obesity‐induced vascular dysfunction associated with NLRP3 inflammasome. We hypothesized that (1) voluntary wheel running would normalize NLRP3 inflammasome activation and IL‐1*β* processing in high‐fat‐diet‐induced obese mouse coronary arterioles; and (2) voluntary running would restore NO signaling and reduce oxidative stress in the coronary arterioles of the obese mice.

## Methods

### Experimental animal model

38 male C57BL/6J wild‐type mice (7 weeks old) were purchased from Jackson Laboratory. The C57BL/6J mouse strain is widely utilized as an obese and CVD model which is easily developed by HFD feeding. All animals were maintained on a 12:12 h light‐dark cycle, controlled temperature and humidity, and given autoclaved water at the animal care facility at the University of Houston. Following 1 week of acclimation, the mice were fed with low‐fat diet (LFD) (4.5% calories from fat, Purina normal rodent chow) or high fat diet (45% calories from fat), and housed in standard mouse cages or in rat cages equipped with a 4.5 inch‐diameter metal running wheel (KATEE Run‐Around Exercise Wheel) fitted with a cycling computer (Sigma BC 8.12) for 12–14 weeks prior to sacrifice. Our simple experimental logic was to utilize voluntary wheel running as a natural type of physical activity to determine primordially preventive effect on high fat diet‐induced obesity, consequently preventing from endothelial dysfunction in the early stage of vascular pathology development. Thus, the voluntary wheel running and the high fat‐diet were initiated simultaneously at the beginning of the experiment. The 45% high fat diet was (Research Diet, D12451) consisting of following energy sources: 20% protein (total diet contained 19.7% casein and 0.3% l‐Cysteine); 35% carbohydrate (total diet contained 17% sucrose, 9.9% maltodextrin, and 7.2% corn starch); 45% fat (total diet contained 39.8% lard and 5.5% soybean oil). All running mice were singly housed in a cage equipped with the running wheel. Voluntary wheel running distances and body weight were measured daily and weekly. All mice were randomly assigned to either low‐fat diet (normal rodent chow) with sedentary (LF‐SED, *n* = 10) as a reference, low‐fat diet with running (LF‐RUN, *n* = 10), high fat diet with sedentary (HF‐SED, *n* = 8), or high‐fat diet with running (HF‐RUN, *n* = 10). Prior to sacrifice at 20–24 weeks of age, whole body composition and abdominal girth were assessed by nuclear magnetic resonance imaging (Echo MRI™‐100H and EchoMRI™‐130; Echo Medical Systems, Houston, TX) and tapeline. Mice were sacrificed using isoflurane and bilateral thoracotomy 24 h after cessation of voluntary running to reduce acute effect of running exercise on subsequent experiment. Isolated cardiac tissue was frozen in liquid nitrogen. All procedures conformed to the approved guidelines set by the Institutional Animal Care and Use Committee at the University of Houston.

### Immunofluorescence

Immunofluorescence experiments were performed as described in our previous study to determine caspase‐1 and IL‐1*β* localization on the endothelium of coronary arterioles with diameters ranging from 50 to 100 *μ*m (Park et al. 2011). The freshly frozen heart was sectioned at 10 *μ*m. After 1 h acetone permeabilization, slides were incubated with blocking solution (10% goat serum in PBS) for 30 min at room temperature after three PBS washes. A mouse on mouse (M.O.M., Vector Laboratories, Burlingame, CA) kit was also used to prevent nonspecific binding between testing tissue and an antibody from mouse host where needed. The sections were then incubated for 1 h (room temperature) in blocking buffer with primary antibodies, including *α*‐smooth muscle actin (SMA, ab5694; Abcam, 1:500), PECAM‐1 (ab24590; Abcam, 1:100), caspase‐1 (ab1872; Abcam, 1:100), IL‐1*β* (ab9722; Abcam, 1:100). After three washes in PBS, sections were incubated with the fluorescence‐conjugated secondary antibody, for example, Alexa Fluor^®^ 488 and Alexa Fluor^®^ 594 (A11001 and A11080; Thermo Scientific) and mounted with ProLong antifade solution (P36930; Thermo Scientific, Waltham, MA). Negative controls were performed with the use of mouse IgG1, mouse IgG, and rabbit IgG (Santa Cruz Biotechnology, Dallas, TX). Images were taken with an Olympus BX41 fluorescence microscope, using Uplan FLN 40× objectives (NA = 1.3). Microscope and camera settings were kept constant throughout the process for each protein assessed. NIH‐ImageJ plug‐in colocalization was used to quantify the IL‐1*β* and caspase‐1 endothelium localization described in the previous study (Lee et al. 2017). All images were converted into 8‐bit gray‐scale images and two points in the images were considered colocalized according to the following criteria: (1) respective pixel intensities (0–255) were greater than 50.0, the threshold of their channels, and (2) the ratio of intensity was higher than the setting ratio (i.e., 50%). The colocalization regions were highlighted with a white overlay mask on the original red and green images and the generated colocalized images were used to analyze area and pixel intensity. Surrounding smooth muscle thickness of coronary arterioles was measured after the images were processed by edge finding with NIH‐ImageJ software.

### Protein expression assessment by western blotting

Hearts were homogenized in RIPA lysis buffer (9806; Cell Signaling). Protein concentration was determined by BCA kit assay (23225; Pierce Biotechnology). An equal amount of protein (30 *μ*g) was separated by sodiumdodecyl sulphate polyacrylamide gel electrophoresis and transferred onto a nitrocellulose membrane (Bio‐Rad). Subsequently, the membrane was blocked with 5% nonfat dry milk or 3% bovine serum albumin (37520; Thermo Fisher Scientific) in TBST for 1 h (room temperature). Primary antibodies for NLRP3 (ab4207; Abcam, 1:500), ASC (sc22514; Santacruz, 1:500), caspase‐1 (ab108362; Abcam, 1:500), IL‐1*β* (ab9722; Abcam, 1:500), eNOS (#32027; Cell Signaling, 1:500), NOX2 (ab129068; Cell Signaling, 1:1000), NOX4 (ab133303; Abcam, 1:5000), and *β*‐actin (sc47778; Santa Cruz Biotechnology, 1:2000) were incubated at 4° overnight, and those were matched to their corresponding horseradish peroxidase‐conjugated secondary antibodies for 1 h (room temperature). Blots were developed with the enhanced chemiluminescence (RPN2232; GE Healthcare Life Science), scanned with the densitometer (FluorChem IS8900; Alpha Innotech, Santa Clara, CA), and quantified using NIH‐ImageJ software. Data were normalized with the corresponding internal reference *β*‐actin. Relative value was normalized to wild‐type control, LF‐SED.

### RT‐qPCR

Total RNA was extracted from cardiac tissue using Trizol reagent (15596; Life Technologies). RNA (2 *μ*g) was treated with DNAse I and reverse‐transcribed into cDNA in a total volume of 40 *μ*L. Real‐time PCR reactions were performed with 12.5 *μ*L of SYBR Green (4309155; Life Technologies) and 2.5 *μ*mol/L of each primer in triplicate using an RT‐qPCR system (ABI Prism 7900HT instrument; Applied Biosystems). Mouse primers used were meNOS, (F) GCTGCACCACAGCAAGCA and (R) AGAATGGTTGCCTTCACACG; and mCyclophilin, (F) GGAGATGGCACAGGAGGAA and (R) GCCCGTAGTGCTTCAGCTT.

Relative mRNA levels were calculated using the double delta CT analysis normalized to cyclophilin, and then compared to LF‐SED (defined as 1.0‐fold).

### NO estimation

Cardiac tissues were deproteinized using Deproteinizing Sample Preparation Kit–TCA (ab204708; Abcam), and the total nitrate/nitrite content was measured with Colorimetric Nitric Oxide Kit (ab65328; Abcam). Samples were run in duplicates following the manufacturer's instruction. The method was based on a two‐step process. The first step converts nitrate to nitrite using nitrate reductase. The second step converts nitrite to a deep purple azo compound using Griess reagents. A colorimetric microplate reader at 540 nm was used to measure the level of azo compounds reflecting the NO amount in the sample.

### Measurement of intracellular superoxide ($O_{2}^{\cdot -}$)

Redox‐sensitive fluorescence dye dihydroethidium (DHE) was used to evaluate $O_{2}^{\cdot -}$ production in coronary arterioles. Frozen cardiac tissue, containing coronary arterioles, cross‐sections were cut 10 *μ*m thick at −25°C in a cryostat (Leica CM 1950, Leica Biosystems Inc., Buffalo Grove, IL). Sections were incubated for 15 min at 37°C under 95% O_2_ and 5% CO_2_ conditions in DHE (D1168; Invitrogen: 10^−5^ mol/L). DHE stained images were visualized by an Olympus BX51 fluorescence microscope and acquired with a 60× objective lens at an excitation peak of 545 nm with an emission spectral peak of 610 nm.

### Statistical analysis

All data are presented as mean ± SEM. SPSS (version 22; IBM, Armonk, NY) was used for all statistical analysis. Running distance between LF and HF were tested using independent *t*‐test following *Levene's Test* for equal variance. All other data were analyzed using One‐way ANOVA followed by LSD post hoc. Statistical significance was accepted at *P *<* *0.05.

## Results

### Effects of voluntary running exercise on HFD‐induced obesity

**Figure 1:**
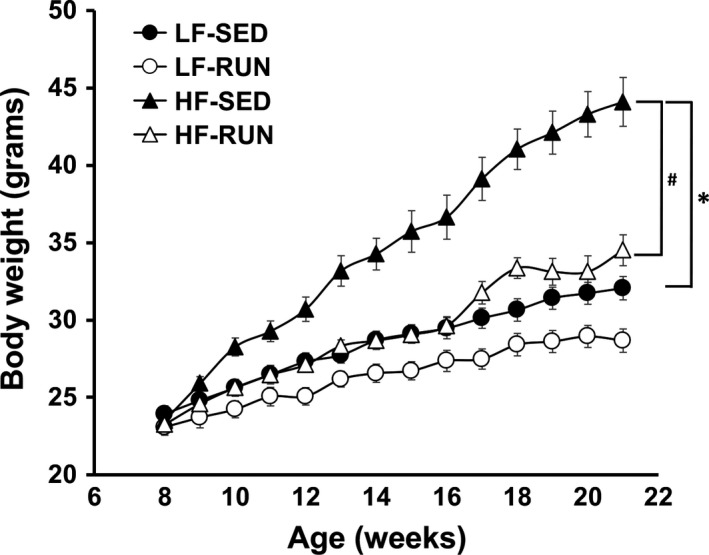
Voluntary running prevents HFD‐induced obesity. Weekly body weight change in response to diet and exercise was recorded for 13 weeks. Voluntary running exercise decreased body weight of mice fed control diet. HFD significantly increased body weight. Voluntary running prevented HFD‐induced development of obesity. Values are means ± SEM. *n* = 8 (HF‐SED) to 10 (LF‐SED, LF‐RUN, and HF‐RUN). *P <* *0.05 versus LF‐SED; ^#^ *P *<* *0.05 versus HF‐SED. HF‐SED, high‐fat diet with sedentary; LF‐SED, low‐fat diet with sedentary; LF‐RUN, low‐fat diet with running; HF‐RUN, high‐fat diet with running.

The table illustrates the effect of HFD or voluntary wheel running exercise for 12–14 weeks. HFD increased body mass, abdominal girth, % body fat, fat mass, and decreased heart to body weight ratio and % lean mass compared to the reference LF‐SED group. LF‐RUN showed a significant reduction in body fat, abdominal girth, % body fat, and % fat mass compared to the LF‐SED group that confirms the effectiveness of the running exercise. Voluntary running initiated with the start of HFD attenuated increase in body mass, % body fat, and fat mass, and it increased heart to body weight ratio and % lean mass. In the running records, HF‐RUN group showed longer running distances and time, and faster average and maximal speed compared to the LF‐RUN group. A weekly increase in body weight is shown in Figure 1. The body weight of HF‐SED was significantly higher than LF‐SED, while voluntary running normalized the weight gain in HFD feeding mice the across study period.

### Inhibitory effect of voluntary running on NLRP3 inflammasome activation in obese heart

**Figure 2:**
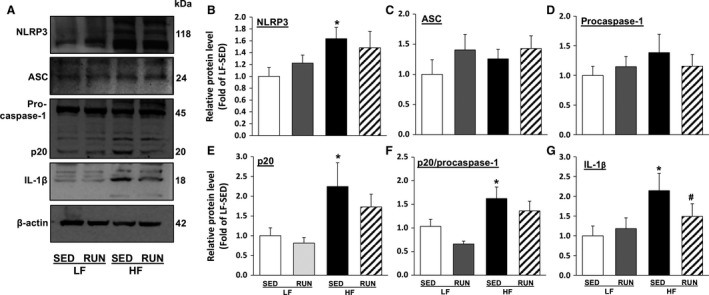
Voluntary running attenuates NLRP3 inflammasome activation in HFD feeding mice heart. Representative western blot images show the inhibitory effect of voluntary running exercise on NLRP3 inflammasome components and IL‐1*β* expression in mouse hearts (A). Summarized data show the effect of HFD or voluntary running on the relative proteins expression levels of NLRP3 (B), ASC (C), inactive procaspase‐1 (D), active caspase‐1 p20 (E), the p20/procaspase‐1 ratio (F), and matured IL‐1*β* (G) in mouse hearts. Values are means ± SEM. *n* = 6–9/group. *P <* *0.05 versus LF‐SED. NLRP3, nucleotide‐binding oligomerization domain‐like receptor family pyrin domain‐containing 3; HFD, high‐fat diet; IL, interleukin; ASC, apoptosis‐associated speck‐like protein containing; LF‐SED, low‐fat diet with sedentary.

The expression of proteins associated with NLRP3 inflammasome was measured to investigate the impact of HFD and voluntary running on NLRP3 inflammasome responses. Compared to LF‐SED, HF‐SED mice had higher expression of NLRP3 (Fig. 2B), cleaved (active) caspase‐1 p20 (Fig. 2E), and IL‐1*β* (Fig. 2G). The ratio of the biologically active form of caspase‐1 p20 to its inactive procaspase‐1 was also increased, indicating increased caspase‐1 activity (Fig. 2F). Voluntary running tended to have an inhibitory effect on HFD‐induced NLRP3 inflammasome expression, that is, reductions of 18% (*P* = 0.181), 23% (*P* = 0.321), and 16% (*P* = 0.156) in NLRP3, caspase‐1 p20, and p20/caspase‐1, respectively (Fig. 2B, E, and F). We found that voluntary running significantly decreased IL‐1*β* in HF‐SED mice (Fig. 2G). There was no effect of HFD or voluntary exercise on ASC and caspase‐1 proteins (Fig. 2C and D). These results demonstrate that HFD induced NLRP3 inflammasome activation in mouse myocardium containing coronary arterioles, and that voluntary running ameliorated its effect.

### Effect of voluntary running on NLRP3 inflammasome‐mediated IL‐1*β* in coronary arteriole endothelium

**Figure 3:**
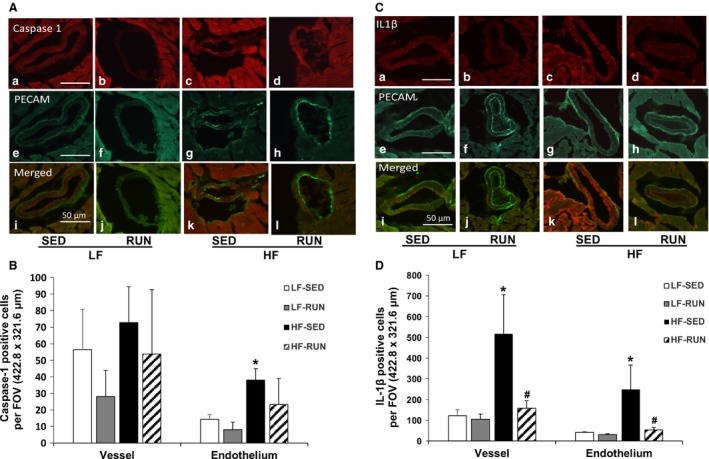
Running exercise reduces expression of caspase‐1 and IL‐1*β* in a coronary vessel. Dual fluorescence combining caspase‐1 (red, a–d) or IL‐1*β* (red, a–d) with PECAM (green, e–h) with the use of specific antibodies against PECAM followed by fluorescent‐labeled secondary antibodies in mouse coronary arterioles (A and C). Merged images (i–l) show the expression of IL‐1*β* (red) or caspase‐1 (red) in coronary arterioles (A and C). Quantification graphs display protein expression of caspase‐1 and IL‐1*β* in the whole coronary vessel and endothelial cells, showing that running exercise reduced HFD‐induced IL‐1*β* expression in coronary vessels and endothelial cells (B and D). Data shown are representative of three separate experiments. *n* = 9 field of view/three animals/group. *P <* *0.05 versus LF‐SED; ^#^ *P *<* *0.05 versus HF‐SED. IL, interleukin; LF‐SED, low‐fat diet with sedentary; HF‐SED, high‐fat diet with sedentary.

Since we found that HFD‐induced activation of the NLRP3 inflammasome in mouse myocardium containing coronary arterioles was blunted by running exercise, we further tested whether decreased NLRP3 is endothelium‐specific. Hence, we performed immunofluorescence to determine the location of HFD‐induced NLRP3 inflammasome activation. First, we confirmed the selectivity of PECAM1 to the specific to the endothelium monolayer by co‐staining *α*‐SMA. We verified that PECAM1 is specific to the endothelium as it aligned at the inner region of SMA (data not shown). We also confirmed that there are no differences in morphology or smooth muscle thickness (8.19 ± 1.47 vs. 9.99 ± 2.08 vs. 9.24 ± 2.21 vs. 8.71 ± 1.05 *μ*m, LF‐SED, LF‐RUN, HF‐SED, HF‐RUN, respectively, *P* = 0.903) between all four groups. Then, we examined whether HFD or voluntary running altered the expression of caspase‐1 and IL‐1*β*, the crucial indicators of NLRP3 inflammasome activation, in a coronary arteriole endothelium of mice. Caspase‐1 and IL‐1*β* were present in the endothelial cells (Fig. 3). While HFD and voluntary running did not significantly change the expression of caspase‐1 in entire coronary arteriole (Fig. 3A and B. Vessel), it was significantly increased by HFD (*P* = 0.047) but the increase was prevented by voluntary running in endothelial cells (Fig. 3A and B. Endothelium). However, IL‐1*β* was also significantly increased in coronary arteriole and endothelial cells of HF‐SED while running exercise diminished IL‐1*β* in coronary arteriole and endothelium of HFD mice (Fig. 3C and D).

### Effects of voluntary running on impairment of NO signaling and increased oxidative stress in HFD‐induced obese heart

**Figure 4:**
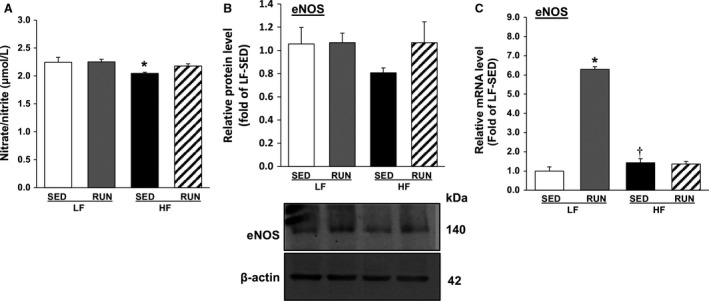
Voluntary running restores NO signal in obese mice heart. NO concentration in mouse heart tissues was analyzed by the content of total nitrate/nitrite followed by a colorimetric microplate reader (*n* = 7–9/group), showing that voluntary running improved decrease in NO contents in HFD feeding mouse cardiac tissues (A). Protein and mRNA levels of eNOS in mouse hearts were assayed by western blot and qPCR analyses, indicating that voluntary running led to a restoring trend HFD‐induced downregulation of protein and mRNA levels of eNOS (B and C). Values are means ± SEM. *n* = 7–8/group. *P <* *0.05 versus LF‐SED; ^†^ *P *<* *0.05 versus LF‐RUN. NO, nitric oxide; HFD, high‐fat diet; eNOS, endothelial nitric oxide synthase; LF‐SED, low‐fat diet with sedentary; LF‐RUN, low‐fat diet with running.

Total nitrate/nitrite, stable metabolites of NO providing an index of NO levels, and eNOS protein and mRNA expression were measured to determine the effect of voluntary running on HFD‐induced alteration of NO signaling. The total absolute nitrate/nitrite concentrations of LF‐SED and LF‐RUN were identical, but were significantly lower in HF‐SED (Fig. 4A). Voluntary running abrogated the HFD‐induced decrease in nitrate/nitrite (by 6.3% in HF‐RUN, *P* = 0.059), so that HF‐RUN did not differ from LF‐SED in NO concentration (Fig. 4A). The protein expression of eNOS in HF‐SED tends to be lower compared to LF‐SED (*P* = 0.051), but it is abolished by voluntary running, which is similar to the levels in LF‐SED (Fig. 4B). The mRNA expression of eNOS was significantly higher in LF‐RUN, but the level did not change within HF‐SED and HF‐RUN (Fig. 4C).

**Figure 5:**
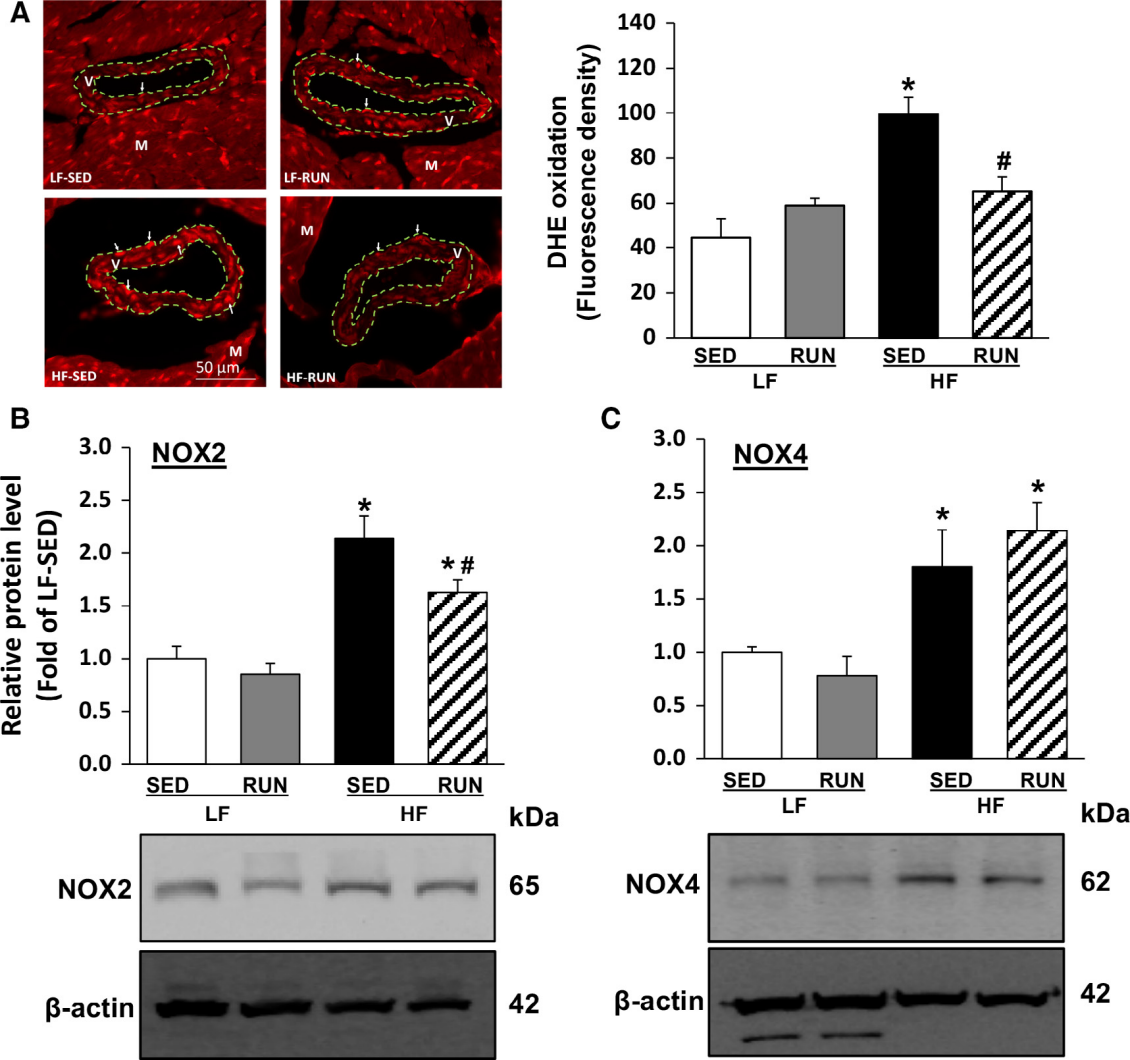
Voluntary running suppresses HFD‐induced oxidative stress in mouse coronary arterioles. DHE fluorescence staining was utilized to measure $O_{2}^{\cdot -}$ production in coronary arterioles and quantified from four independent experiments using the different sample (2–3 coronary arterioles/heat, *n* = 4/group), showing HFD increased coronary $O_{2}^{\cdot -}$ production while it was attenuated by running exercise (A). Cardiac protein expression levels of NOX2 (B) and NOX4 (C) were analyzed by western blot to investigate molecular mechanisms for $O_{2}^{\cdot -}$ production. Values are means ± SEM. *n* = 7–8/group. *P <* *0.05 versus LF‐SED; ^#^ *P *<* *0.05 versus HF‐SED. HFD, high‐fat diet; DHE, dihydroethidium; LF‐SED, low‐fat diet with sedentary.

The effects of voluntary running on oxidative stress in coronary arterioles of obese mice were assessed by measuring the level of superoxide ($O_{2}^{\cdot -}$), and expression of cardiac NOXs. DHE fluorescence imaging of $O_{2}^{\cdot -}$ in mouse coronary arterioles and its quantification (Fig. 5A) showed that oxidative fluorescence (white arrow) increased in a coronary arteriole (V, dotted green line) of HF‐SED, but voluntary running reduced oxidative fluorescence in HF‐RUN. As the primary sources of NADPH‐derived $O_{2}^{\cdot -}$ generation in mitochondria and in membrane of the vascular endothelial cells, NOX2 and NOX4 protein expression were analyzed (Fig. 5B and C). HFD increased protein expression of both NOX2 and NOX4. Voluntary running decreased the upregulation of NOX2 in HF‐RUN (Fig. 5B).

## Discussion

The purpose of this study was to determine whether voluntary running exercise can prevent (1) coronary NLRP3 inflammasome activation and (2) impaired NO signaling and enhanced oxidative stress in high‐fat‐diet‐induced obese mice. The major findings of this study are as follows: (1) voluntary running reduced the expression of caspase‐1 and IL‐1*β* in coronary arteriolar endothelium of obese mice and (2) voluntary running restored NO production and reduced oxidative stress in HFD mice. The findings suggest that voluntary running suppresses NLRP3 inflammasome activation and oxidative stress, and enhances NO production, and that these benefits have association with prevention of vascular dysfunction in obesity.

### Voluntary running effect on HFD‐induced obesity

In this study, we demonstrated that consumption of HFD developed obesity, and that voluntary wheel exercise protected HFD‐induced alterations in body composition, including body mass, abdominal girth, % body fat, fat mass, and heart mass to body mass ratio (Table 1). Our results are consistent with previous studies using a primordial intervention that voluntary running provides protective effects on obesity by inhibiting fat accumulation and preserving lean mass (Park et al. 2012; Yan et al. 2017). While plasma insulin and glucose levels are absent in this study, our previous study (Park et al. 2012) using the same mice and modality of physical activity and diet showed a significantly higher plasma insulin level in HF‐SED compared to the one in LF‐SED and in HF‐RUN. Thus, it is assumed that voluntary running would return the increased insulin level in the HFD to the level in LF‐SED.

**Table 1:** Effect of high‐fat dietary feeding and voluntary wheel running on obesity

	LF		HF	
	SED (*n* = 10)	RUN (*n* = 10)	SED (*n* = 8)	RUN (*n* = 10)
Final body mass, g	33.04 ± 0.53	29.37 ± 0.68	45.89 ± 1.57 a	36.64 ± 0.91 a ^,^ b
Heart mass, mg	158.18 ± 5.78	155.93 ± 6.57	156.26 ± 6.82	159.43 ± 4.90
Heart to body mass ratio, mg/g	4.8 ± 0.23	5.3 ± 0.21	3.4 ± 0.20 a	4.4 ± 0.18^b^
Abdominal girth, cm	8.17 ± 0.14	7.49 ± 0.14 a	9.74 ± 0.26 a ^,^ c	8.58 ± 0.17
% body fat, %	18.46 ± 1.77	13.53 ± 0.59 a	39.69 ± 2.66 a ^,^ c	29.70 ± 1.71 a ^,^ b ^,^ c
Fat mass, g	6.12 ± 0.62	3.97 ± 0.19 a	18.46 ± 1.68 a ^,^ c	11.01 ± 0.89 a ^,^ b ^,^ c
% lean mass, %	74.62 ± 1.76	78.36 ± 0.47	55.00 ± 2.51 a ^,^ c	63.41 ± 1.53 a ^,^ b ^,^ c
Lean mass, g	24.62 ± 0.56	23.02 ± 0.58	25.00 ± 0.58	23.12 ± 0.19^b^
Running distance, km/d	–	9.23 ± 1.22	–	13.06 ± 1.11^c^
Running time, min	–	265 ± 12	–	387 ± 31^c^
Average speed, km/h	–	1.68 ± 0.03	–	1.89 ± 0.04^c^
Maximum speed, km/h	–	3.23 ± 0.06	–	3.41 ± 0.03^c^

Values are means ± SEM. LF, low‐fat diet; HF, high‐fat diet; SED, relatively sedentary lifestyle; RUN, voluntary wheel running. All groups are represented as LF‐SED, LF‐RUN, HF‐SED, and HF‐RUN using combinations of different diets and activities.

a *P *<* *0.05 versus LF‐SED.

b *P *<* *0.05 versus HF‐SED, *n* = 8 (HF‐SED) to 10 (LF‐SED, LF–RUN, and HF‐RUN).

c *P *<* *0.05 versus LF‐RUN.

Unexpectedly, we found that HF‐RUN did not reach the same level of body weight, % body fat, and body fat mass as LF‐SED despite a significant decrease in these markers (Table 1). We note that our previous study found that voluntary running completely reversed HFD‐induced increases in body weight and body fat in female C57BL/6J mice (Park et al. 2012). The previous studies reported that female mice with the same strain ran longer distance on the voluntary running wheel than the corresponding male mice (De Bono et al. 2006) and that HFD male mice tended to have more dramatic weight gain than female mice with identical diet (Yang et al. 2014). Therefore, we speculate that male mice tend to have less activity during voluntary running while more body weight gain by HFD compared to female mice.

Notably, our voluntary running mice fed HFD ran longer distances (13.06 ± 1.11 km) compared to LF‐RUN (9.23 ± 1.22 km). There is no direct study to explain this phenomenon, yet a previous study reported increased daily running distance at the beginning of HFD and end of HFD period in same strain female mice (Basterfield et al. 2009). Importantly, there was higher total activity in HFD running group compared to LFD running group (Basterfield et al. 2009). The obese mice with insulin resistance due to high fat and high sucrose diet also exhibited increased wheel‐running distance compared to the control diet mice group (Meek et al. 2012).

Therefore, there might be defensive mechanism counteracting diet‐induced obesity by regulating physical activity. Future studies to investigate more detail information about physical activity (speed and duration), energy expenditure (basal vs. exercise), and neural cycle during HFD.

### Effects of voluntary running on vascular NLRP3 inflammasome activation and IL‐1*β* processing in obese mice

Previous studies showed that exercise training attenuates NLRP3 inflammasome activation in ovariectomized mouse brain hippocampus (Wang et al. 2016b) and in HFD‐induced obese mouse adipose tissues (Mardare et al. 2016). However, no study has investigated the effects of physical activity on coronary vascular NLRP3 inflammasome in diet‐induced obesity. We report here for the first time that exercise training reduced the enhanced protein expression of NLRP3, active caspase‐1 p20, a ratio of active caspase‐1 p20 to inactive procaspase‐1, and matured IL‐1*β* in hearts (Fig. 2) and in coronary arterioles (Fig. 3) of HFD‐induced obese mouse. A possible cause for the lack of statistical significance to show the exercise effect in Figure 2 is the use of cardiac tissues, rather than isolated coronary arterioles in western blotting due to an insufficient amount of the vessels. This explanation is supported by the immunofluorescent results localizing caspase‐1 and IL‐1*β* specifically in coronary arteriole endothelium (Fig. 3). These demonstrate that voluntary running normalizes HFD‐induced increase in caspase‐1 and IL‐1*β*, the most representative indicators of NLRP3 inflammasome activation, in coronary endothelial cells (Fig. 3). Recent studies have emphasized the critical role of caspase‐1 in endothelial injury to initiate endothelial dysfunction (Zhang et al. 2015; Xi et al. 2016). This study provides the direct evidence that physical activity is an effective strategy to attenuate HFD‐induced NLRP3 inflammasome signaling, including caspase‐1 cleavage‐dependent vascular injury and IL‐1*β* processing, which is associated with vascular dysfunction in coronary microcirculation. The future study measuring the vascular reactivity using the inhibitors of NLRP3 and caspase‐1 can determine the direct roles of inflammasome in obesity‐associated vascular dysfunction and the impact of physical activity.

Regarding the suppressive effect of voluntary running exercise on NLRP3 inflammasome activation in obesity, we propose several possible inhibitory pathways. First is the exercise‐induced reduction in excessive endogenous danger signals, such as free fatty acids, cholesterol, ATP, ROS, etc., that initiate the transcription of NLRP3 inflammasome components and the subsequent activation of the inflammasome (Ringseis et al. 2015). The current results demonstrate that voluntary running reduces $O_{2}^{\cdot -}$ production in coronary arterioles of HFD mice (Fig. 5A) building on our previous study using the same primordial prevention concept which reported that chronic voluntary running normalizes plasma insulin, glucose, and $O_{2}^{\cdot -}$ generation in HFD (Park et al. 2012). Second, an increase in ketone metabolite *β*‐hydroxybutyrate, known to be released during prolonged or intensive exercise, has been considered as a potential endogenous antagonist of NLRP3 inflammasome components (Youm et al. 2015). Although we did not measure this metabolite in this study, it will be of interest for future studies to include. The third possible inhibitory pathway is NO molecule‐induced suppression of NLRP3 inflammasome activation (Mishra et al. 2013). Impairment of NO production in obesity has been well established, and our data show that the impairment was reversed by voluntary wheel running (Fig. 4). Further experiments, for example, laminar shear‐stress to mimic the exercise effect at the cellular level, are needed to confirm the effect of exercise on NLRP3 inflammasome pathways in vascular function in obesity.

### Possible underlying mechanisms by which chronic voluntary running preserves obesity‐induced vascular dysfunction through NO and oxidative stress pathway

Decreased NO production is a primary indicator of vascular endothelium‐dependent vasodilatory dysfunction. It is governed by the down‐regulation of eNOS activity responsible for producing NO from l‐arginine, the biological precursor of NO, in the vascular endothelial cells (Sena et al. 2013). We found that HFD reduced nitrate/nitrite concentration, which is rapidly oxidized from NO, (Fig. 4A) and eNOS expression (Fig. 4B, 32% lower vs. LF‐SED, *P* = 0.051) in mouse cardiac tissues. Gamez‐Mendez et al. (2015) have reported that HFD treatment reduced coronary NO production and endothelial dysfunction in obese mice, and Yang et al. (2007) have shown a down‐regulation of aortic eNOS expression in HFD‐induced obese rats. Previously, we have demonstrated that HFD impaired NO‐mediated coronary arteriolar vasodilatation in obese mice (Park et al. 2012). This study provides further evidence that diet‐induced obesity diminishes the NO signaling pathway associated with impaired the coronary microcirculation.

The molecular mechanism for the beneficial effects of chronic endurance exercise in the vascular system includes activation of the Akt/PI3K/eNOS signaling pathway by continuous pulsatile blood flow‐mediated laminar shear stress on vascular endothelial cells (Tzima et al. 2005). We found that running mice fed HFD showed a restoring trend in NO production (Fig. 4A, 6.3% higher vs. HF‐SED) and eNOS expression (Fig. 4B, 38% greater vs. HF‐SED) close to the levels seen in LF‐SED. These results support previous reports in the literature. For example, Ito et al. (2015) found that running exercise improved a reduced formation of nitrate/nitrite metabolites in renal cortex and outer medullar of diabetic Zucker rats. Silva et al. (2016) reported that treadmill exercise training reversed downregulation of eNOS protein expression in the aorta of high‐sugary diet‐induced obese mice. Together these results suggest that voluntary running can restore NO signaling in the coronary microcirculation of diet‐induced obese mice. Unlike our previous study reporting that voluntary running protected against HFD feeding‐induced coronary vascular dysfunction through an improved phosphorylation of eNOS (p‐eNOS: ser1177, activation site for NO synthesis) (Park et al. 2012), in this study, we found no significant reduction in p‐eNOS in HF‐SED and its restoration in HF‐RUN (data not shown). We attribute these findings to the length of HFD and voluntary running (8 weeks in the earlier study vs. 12–14 weeks in this study). Sex differences may also account for the differences, as our earlier study employed female mice (Park et al. 2012). Yang et al. (2014) reported that 35 weeks of HFD resulted in greater body weight gain (71% increase) in male C57BL/6J mice compared to in female mice (13% increase), suggesting that male mice experienced greater attenuation of phosphorylation of eNOS. A discrepancy between mRNA and protein expression of eNOS is possibly due to physically active nature of mice model. Running on voluntary wheel was only considered as physical activity in this study, but baseline behavioral activity, such as locomotor activity and grooming, was not measured. Increased eNOS mRNA expression of LF‐RUN did not change level of eNOS protein, speculating that excessive eNOS expression beyond normal physiological state to maintain homeostasis might be unnecessary.

An increase in NADPH oxidase‐dependent ROS production in obesity is involved in the down‐regulation of the NO signaling pathway, and contributes to pathologies of endothelial dysfunction. We found that HFD significantly increased $O_{2}^{\cdot -}$ production in coronary arterioles, and that voluntary running negated diet‐induced increase of $O_{2}^{\cdot -}$ production (Fig. 5A). This finding indicates that voluntary running could prevent an HFD‐induced elevation of ROS production. To ascertain molecular mechanism(s) contributing to $O_{2}^{\cdot -}$ production, we assessed NOX2, the major source of NADPH oxidase activity‐derived $O_{2}^{\cdot -}$ generation in endothelium (Konior et al. 2014), and NOX4, which is known to predominantly produce hydrogen peroxide (H_2_O_2_) and act as endothelium‐dependent hyperpolarizing factor inducing vasodilation rather than $O_{2}^{\cdot -}$ generation (Ray et al. 2011). Our results showing that HFD treatment resulted in up‐regulation of NOX2 and NOX4 proteins in mouse cardiac tissues (Fig. 5B and 5C) are consistent with existing evidence that obese and diabetic conditions enhance NADPH oxidase activity (Gao et al. 2007; Park et al. 2012). Our finding that voluntary running reduced diet‐induced up‐regulation of NOX2 protein expression (Fig. 5B) is consistent with our previous study that running exercise reversed the increased protein expression level of gp91phox (NOX2) (Park et al. 2012). However, we note that voluntary exercise did not alter the HFD‐induced NOX4 elevation (Fig. 5C); consistent with this finding, Novoa et al. (2017) found that exercise did not change the level of mRNA NOX4 in diabetic rat cardiac tissues. Thus, while exercise might not affect regulation of NADPH subunit NOX4 in the cardiovascular system, further investigation is needed to identify the exact mechanisms. Our overall results provide evidence that chronic endurance exercise has protective effects, such as anti‐obesogenic, anti‐inflammation, and redox and NO signaling homeostasis, on HFD consumption‐induced coronary vascular dysfunction.

A limitation of this study is the use of cardiac tissue, containing coronary vessels, were utilized to perform western blotting and RT‐qPCR. This is mainly due to the small amount of coronary arteriole available from the mice heart. To explore the specific expression of caspase‐1, IL‐1*β* and superoxide in the coronary arteriolar endothelium, immunofluorescence of frozen cardiac section was utilized.

## Conclusion

Our study provides evidence that a primordial prevention strategy of voluntary wheel running can prevent major risk factors of cardiovascular pathology in mice of obesogenic environment: (1) ameliorating NLRP3 inflammasome activation which leads to caspase‐1‐dependent‐IL‐1*β* processing; and (2) restoring impaired NO bioavailability and $O_{2}^{\cdot -}$‐associated vascular dysfunction. We suggest that regular physical activity is an effective strategy to protect diet‐induced vascular dysfunction by suppressing NLRP3 inflammasome activation and possibly improving NO bioavailability.

## Conflict of Interest

The authors have no conflicts of interests to disclose. The results of this study are presented clearly, honestly, and without fabrications, falsification, or inappropriate data manipulation.
